# Absorbing it all: A meta-ethnography of parents’ unfolding experiences of newborn screening

**DOI:** 10.1016/j.socscimed.2021.114367

**Published:** 2021-10

**Authors:** Ashley L. White, Felicity Boardman, Abigail McNiven, Louise Locock, Lisa Hinton

**Affiliations:** a Nuffield Department of Primary Care Health Sciences, University of Oxford, Oxford, UK; b Warwick Medical School, University of Warwick, Coventry, UK; c Health Services Research Unit, University of Aberdeen, Aberdeen, UK; d The Healthcare Improvement Studies Institute, University of Cambridge, Cambridge, UK

**Keywords:** Meta-ethnography, Qualitative research, Newborn screening

## Abstract

In a context of increasing international dialogue around the appropriate means and ends of newborn screening programmes, it is critical to explore the perspectives of those directly impacted by such screening. This meta-ethnography uses a systematic review process to identify qualitative studies that focus on parents' experiences of newborn screening published in English-language academic journals from 2000 to 2019 (n = 36). The included studies represent a range of moments, outcomes, and conditions that illuminate discrete elements of the newborn screening journey. We draw on these varied studies to construct a diagram of possible newborn screening pathways and through so-doing identify a critical window of time between the signalling of a positive newborn screen and the end of the screening process. During this critical window of time, families navigate complex emotional reactions, information, and decisions. From an in-depth analysis of this data, we develop the concept of “absorptive capacity” as a lens through which to understand parents' responses to new and emerging information. Alongside this, we identify how the “concertinaing of time” – the various ways that parents experience the expansion and compression of time throughout and beyond the screening pathway – affects their absorptive capacities. This study underscores the need to move away from viewing newborn screening as a discrete series of clinical events and instead understand it as a process that can have far-reaching implications across time, space, and family groups. Using this understanding of screening as a starting point, we make recommendations to facilitate communication and support for screened families, including the antenatal provision of information to parents and accommodations for the fluctuations in parents’ absorptive capacities across the screening trajectory.

## Highlights

• Parents may not realise the potential ramifications of newborn screening (NBS).
• There is a critical window of time between positive NBS results and further testing.
• “Absorptive capacity” can describe how parents comprehend screening information.
• NBS can have long-lasting implications across time, space and family groups.

“I don't see there is a problem, I don't see what difference it's going to make, it is only a test, it is not gonna change their life or anything is it?“- Mother of a child with screen-negative newborn screening results (Parsons et al., 2007, 62).

## 1 Introduction

The rapid development of genomic technologies in recent years has brought newborn screening policies under renewed, and international, scrutiny. Technologies such as CRISPR-Cas9, with their potential to relatively quickly and cheaply generate high output mass screens through whole-genome/exome sequencing, has triggered a re-imagining of the purposes and boundaries of newborn screening practices on a scale not previously witnessed. Indeed, across the globe, a number of pilot studies have emerged in recent years exploring the value and feasibility of whole-genome sequencing as a population screening tool, including large-scale projects in Australia (Mackenzie’s Mission, 2019), the United States ("The BabySeq Project," 2020), and the United Kingdom (Siden, 2019).

Newborn screening was initially introduced in the United Kingdom in the 1950s as a means to identify infants with the metabolic disorder phenylketonuria (PKU), a condition for which early identification and intervention has a significant impact on outcomes for affected children. In 1968, Wilson & Jungner laid out ten principles for assessing the validity of population screening programmes. Among these included the statement, “Of all the criteria that a screening test should fulfil, the ability to treat the condition adequately, when discovered, is perhaps the most important” (Wilson and Jungner, 1968, 26). Since then, the newborn bloodspot screen (or “heel prick”) has expanded to include multiple conditions to varying extents across countries. In the United Kingdom, which has traditionally adopted a conservative approach to newborn screening relative to other countries (Downing and Pollitt, 2008), the newborn bloodspot screen currently includes only a further eight conditions, the majority of which are inherited metabolic conditions. Conditions currently screened for include: PKU, sickle cell disease, cystic fibrosis (CF), congenital hypothyroidism, medium-chain acyl-CoA dehydrogenase deficiency (MCADD), maple syrup urine disease (MSUD), isovaleric acidaemia, glutaric aciduria type 1, and homocystinuria (pyridoxine unresponsive). Strict adherence to Wilson-Jungner criteria in the evaluation of newborn screening programmes in the United Kingdom explains much of this conservatism, as well as the challenges of accurately weighing the relative harms and benefits (Taylor-Phillips et al., 2014). The United Kingdom's approach is in stark contrast to that used in the United States where, in some states, upwards of 60 conditions are currently screened for (Baby’s First Test, 2020). However, in light of new genomic technologies, these criteria are themselves facing international critique and calls for revision given the unique and unprecedented challenges presented by genomic screening (Andermann et al., 2011; Dobrow et al., 2018).

It is in this context of increasing international dialogue around the appropriate means and ends of newborn screening policies that the necessity of exploring the perspectives of those directly impacted by such screening is now critical. The role of lived, patient experience evidence is increasingly recognised in policy and guideline development (Sheard et al., 2019, Staniszewska et al., 2010). It is only through a systematic analysis of this research evidence that the “real world” impacts of newborn screening, as conceptualised and prioritised by the families who have directly experienced it, can be adequately explored, and represented, in these emergent debates.

It has been argued that qualitative research on newborn screening experiences has been marginalised in debates around screening in favour of quantitative economic and clinical meta-analyses (Grob, 2019). To overlook these perspectives risks impoverishing our understandings of these debates, as qualitative methodologies have the advantage of being able to capture the complexity, and nuance, of patient experience that is often missed by reliance on quantitative methods alone (Sheard et al., 2019). Moreover, qualitative research may allow for the identification of the dimensions of the screening experience most significant to patients, or parents, rather than those prioritised by researchers and clinicians.

Whilst previous qualitative reviews of newborn screening evidence have focused on tightly defined aspects of the screening experience, such as parents’ communication of carrier results to children as they age (Ulph et al., 2014), there have been few attempts to characterise the critical features of the whole newborn screening journey from the family perspective. Such a review is essential to contextualise emerging economic and clinical analyses of newborn screening, and more accurately identify its relative harms and benefits – a key component of screening policy reviews. Given this identified gap in the literature, this paper presents a qualitative synthesis of the newborn screening literature from the past 20 years, bringing together a comprehensive and complex evidence base on newborn screening to facilitate its inclusion and evaluation in newborn policy reviews.

## 2 Methods

This qualitative evidence synthesis was conducted as part of a larger study exploring the measurement of harms and benefits of antenatal and newborn screening programmes in the United Kingdom (Assessing the benefits and harms of antenatal and newborn screening programmes in health economic assessments, 2020). While the larger project is solely focused on the United Kingdom, we reviewed the international literature to interpret existing findings and develop a fuller conceptual understanding of how newborn screening is experienced. We approached the review sensitised to the possibility that different contexts will impact the screening experience but sought to identify cross-cutting themes that transcended these differences.

### 2.1 Approach to meta-synthesis

The goal of a meta-synthesis is to bring together, examine, and interpret findings from disparate qualitative research studies and produce a more in-depth understanding than is possible from looking at the studies individually (Bearman and Dawson, 2013; Finfgeld, 2003). It offers the opportunity to identify patterns, processes, and contexts as well as omissions from a body of work (Erwin et al., 2011). While there are multiple ways synthesising qualitative research (see Barnett-Page and Thomas, 2009), we followed the stages of meta-ethnography as described by Toye et al. (2014). We selected this approach over others such as narrative review (Greenhalgh et al., 2018) because it would allow us to interpret disparate studies and build a line-of-argument synthesis that gives a broader understanding of parents’ experiences with newborn screening. The methodological approach gives room to develop explanatory concepts that translate across studies, and which may be of use to other scholars in the future.

### 2.2 Stages of the meta-ethnography

#### 2.2.1 Getting started

Our research team consists of social scientists with backgrounds in bioethics, medical sociology, public health, and social demography who have extensive combined qualitative research experience.

#### 2.2.2 Searching and sifting the evidence

We wanted to bring together qualitative studies that explored parents’ experiences of newborn screening. We opted to conduct a systematic literature search to a) adhere to the norms of existing research culture, b) provide evidence that we sought to capture as much of the evidence within the scope of our research question as possible, and c) counter claims that qualitative synthesis work is not rigorous (Toye et al., 2014). Recognising that it can be challenging to locate qualitative research studies, we began with a broad, systematic search strategy (Ludvigsen et al., 2016). We searched for any instance of the terms “newborn screen*“, “neonatal screen*“, or “newborn bloodspot” in the title or abstract of academic journals published in English from January 2000 until December 2019 across five databases [see Table 1].

**Table 1:** Search strategy^a^.

Search terms in title or abstract	Databases
Newborn screen∗	CINAHL Complete
Neonatal screen∗	JSTOR
Newborn bloodspot	PsychInfo
	Sociological Abstracts
	Web of Science

a Articles published in English from January 2000 until December 2019.

**Fig. 1:**
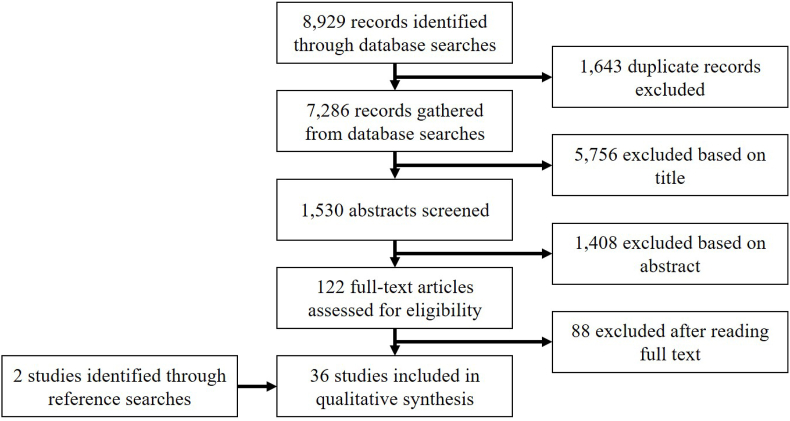
Flow diagram of included and excluded studies.

After running the search, AW removed duplicate records and reviewed titles and abstracts for eligibility. If it was unclear whether or not a record should be included based on the title alone, it remained in the pool until the next step. Next, AW read the remaining studies in their entirety to assess eligibility. LH provided a secondary ruling for articles that AW was uncertain about. As the final step, AW hand searched the reference lists of included studies to identify additional research not identified through the search strategy. AW maintained a database of all decisions about inclusion and exclusion [see Fig. 1].

Studies were eligible for inclusion if they focused on parental experiences of newborn screening programs and used qualitative methods. Non-research publications, such as commentaries or letters to editors, were excluded. Similarly, mixed-methods research was excluded as the qualitative analysis was often secondary to reporting statistical findings. Because we were interested in how parents experienced varying aspects of the newborn screening process, studies that included other stakeholders (e.g., genetic counsellors or midwives) alongside parents were excluded, as it was challenging to separate findings between stakeholder types. We also excluded studies that focused on concerns about storing newborn blood spot cards, experiences of antenatal genetic counselling, experiences of being diagnosed with a screened-for condition in later life, or experiences of living with a screened-for condition. We were unable to include books in this review due to resource constraints; however, scholars who have written books tend to distil their findings in journal articles, too. For example, findings from books by Grob (2011) and Timmermans and Buchbinder (2012) are (partially) accounted for in this review (Grob, 2006, 2008; Buchbinder & Timmermans, 2011a, 2011b, 2012; Timmermans and Buchbinder, 2010).

#### 2.2.3 Quality assurance

We acknowledge that there are conflicting ways of assessing quality in qualitative syntheses (Dixon-Woods et al., 2007). We did not exclude any eligible studies based on quality because of these varying conceptions of how to evaluate qualitative research (Sandelowski et al., 1997). Instead, we recognise that authors were writing with different purposes for different audiences. In some cases, authors were health care providers looking to descriptively explain how patients experienced newborn screening and influence care provision. In others, ethnographers have theorised the meanings attached to newborn screening as a means of contributing to the sociological literature. Papers included in this synthesis reflect the wide-ranging disciplinary backgrounds and purposes of the authors.

#### 2.2.4 Data extraction

Once we had a finalised list of included studies, we divided the work of reviewing amongst the research team. AW read all of the studies and maintained an excel database tracking study characteristics. We split the number of studies (n = 36) evenly amongst the remaining four members of the research team so that each individual was responsible for focusing on nine studies. By using this approach, we sought to ensure each study received appropriate consideration. We read the included studies in their entirety, with particular attention focused on the findings and discussion sections of studies. We used a combination of computer-aided and paper-based reading and coding processes. AW uploaded PDFs for the included studies to NVivo 12 Pro. She progressed from a line-by-line coding approach to organising codes into descriptive themes and then refining codes into conceptual categories. The remaining researchers used a paper-based approach to coding, where they read the studies and hand-coded higher-level concepts as they emerged. They made a note of these concepts and shared them with the research team.

#### 2.2.5 Data synthesis

After we analysed the studies individually, we set about synthesising the literature and identifying meta-themes. This was an iterative process that took place over several months. We arranged evidence synthesis meetings to discuss our analysis and identify cross-cutting themes. We paid particular attention to the concepts, ideas, and phrases used in different studies. Ultimately, we included both the words of participants as reported in studies, as well as the interpretations offered by the authors of the studies themselves in our analysis. As we moved forward with our analysis, we also considered the ways that concepts could be applied across studies. This was an iterative process where individuals generated overarching concepts based on the papers that they had read and brought these ideas to the group collaboration meetings. We discussed these overarching concepts and considered how they might apply to various other studies in the synthesis. Even though individuals were responsible for different subsets of studies, we generally found that we had developed overlapping concepts, although we might have called them different names.

#### 2.2.6 Developing the findings

As we reached consensus on the core experience dimensions drawn from the compiled literature, we synthesised these findings into higher-order interpretations that prevailed across screening contexts and conditions. In this way, the synthesis can make a statement on what qualitative research studies have found about the experiences and implications of newborn screening.

#### Ethical approval

This review did not require ethical approval since it drew on existing publications.

## 3 Findings

### 3.1 Mapping newborn screening pathways from included studies

Our systematic review yielded a total of 36 studies [see Table 2]. The majority of studies were conducted in the United States (n = 18) or the United Kingdom (n = 12), with the remaining coming from Australia (n = 2), Israel (n = 2), or New Zealand (n = 2). While the studies covered a range of screened-for conditions, cystic fibrosis was the condition most commonly addressed (n = 16). Studies ranged from descriptive accounts of the newborn screening experience to theory generation about the meanings attached to those experiences. Given the complexity of newborn screening, many papers were only able to focus on discrete points in the pathway rather than the whole “journey”. However, through a cross-study analysis, a broader, richer picture takes shape compared to what can be provided in the individual papers.

**Table 2:** Characteristics of included studies (n = 36).

	Author (date)	Date	Country	Condition(s) addressed	Research aim	Participants	Data
1	Boyse et al. (2014)	2014	United States	Congenital adrenal hyperplasia (CAH)	To characterise the experiences and expressed needs of parents following diagnosis of their newborn with congenital adrenal hyperplasia (CAH).	Parents of children diagnosed with CAH (n = 6)	Individual interviews
2	Buchbinder and Timmermans (2011a)	2011	United States	MCADD	To examine how parents and clinical staff work out the social significance of uncertain newborn screening results.	Representative case study of one family with positive newborn screen for MCADD	Ethnographic observation, individual interviews
3	Buchbinder and Timmermans (2011b)	2011	United States	Metabolic conditions	To explore the potential for newborn screening to diagnose mothers with genetic disorders, requiring a reconceptualisation of traditional views of family “beneﬁt”.	Parents of newborn screening patients (n = 7 families)	Ethnographic observation, individual interviews
4	Buchbinder and Timmermans (2012)	2012	United States	Metabolic conditions	To explore parents' perceptions of the initial communication of newborn screening results.	Parents of newborn screening patients (n = 75 families)	Ethnographic observation, individual interviews
5	Carpenter et al. (2018)	2018	United Kingdom	PKU	To explore the experiences of parents of children with PKU under the age of two.	Parents of children with PKU under the age of 2 (n = 7)	Individual interviews
6	Chudleigh et al. (2016)	2016	United Kingdom	Cystic fibrosis or sickle cell disease	To explore parents' experiences of receiving the initial positive newborn screening result for their child with cystic fibrosis or sickle cell disease.	Parents whose children had been diagnosed with cystic fibrosis or sickle cell disease and were less than 1 year old at time of interview (n = 22)	Individual interviews
7	DeLuca et al. (2011)	2011	United States	Metabolic conditions	To describe parents' experiences with testing for rare metabolic conditions.	Parents of children undergoing testing for metabolic conditions (n = 44); 9 children with positive diagnoses, 8 negative, 13 equivocal confirmatory results	Longitudinal interviews during and after metabolic testing process
8	Dillard and Carson (2005)	2005	United States	Cystic fibrosis	To identify how family members communicatively manage the uncertainty created by a positive newborn screening result.	Families of children who had a positive newborn screening test result for cystic fibrosis (n = 17)	Video recordings of medical interactions with families
9	Grob (2006)	2006	United States	Cystic fibrosis	To examine parents' experiences of newborn screening.	Parents of children who received genetic diagnoses via newborn screening for cystic ﬁbrosis (n = 25)	Individual interviews
10	Grob (2008)	2008	United States	Cystic fibrosis	To examine parents' experiences of newborn screening.	Parents of children who were diagnosed with cystic ﬁbrosis either via newborn screen (n = 16); prenatally (n = 4); or after the development of symptoms (n = 15)	Individual interviews
11	Johnson et al. (2019)	2019	United Kingdom	Cystic fibrosis	To explore the psychological impact of receiving a “cystic fibrosis screen positive, inconclusive diagnosis” (CFSPID) result on parents.	Parents of children who received CFSPID (n = 8)	Individual interviews
12	Kerruish (2011)	2011	New Zealand	Type 1 diabetes	To explore the psychosocial impact of screening newborns for genetic susceptibilities using type 1 diabetes as an example of a common disorder with multiple signiﬁcant genetic contributors to its aetiology.	Parents of children who had received increased risk results in a study that involved newborn screening for genetic susceptibility to type 1 diabetes (n = 11)	Individual interviews
13	Kerruish (2016)	2016	New Zealand	Type 1 diabetes	To explore the later effects of screening for genetic susceptibility to a single, complex disorder: type 1 diabetes.	Parents of children who had been tested for genetic susceptibility to type 1 diabetes 12 years previously (n = 15)	Individual interviews
14	Locock and Kai (2008)	2008	United Kingdom	Sickle cell, thalassaemia, other haemoglobin variants	To explore parents' experiences and attitudes towards antenatal and newborn screening for haemoglobin disorders.	Parents who had experienced gene-carrier identification through antenatal and newborn screening for sickle cell, thalassaemia, and other haemoglobin variants within the previous 2 years (n = 39)	Individual interviews
15	Moran et al. (2007)	2007	United Kingdom	Cystic fibrosis	To investigate the emotional impact of false-positive diagnoses.	Parents who received false-positive IRT cystic fibrosis test result (n = 21)	Individual interviews
16	Nicholls (2010)	2010	United Kingdom	None	To highlight differences between parental knowledge of newborn screening and their understanding of what actually took place.	Parents whose children had newborn screening tests (n = 18)	Individual interviews
17	Nicholls (2012)	2012	United Kingdom	None	To explore parents' experiences with the newborn screening consent process.	Parents who had children born in the prior 2 years (n = 18)	Individual interviews
18	Nicholls and Southern (2013)	2013	United Kingdom	None	To understand the factors that influence parental decisions in accepting newborn screening and roles they play in the process.	Parents who had children born in the prior 2 years (n = 18)	Individual interviews
19	Parsons et al. (2007)	2007	United Kingdom	None	To explore mothers' experiences with newborn screening.	Mothers who were offered newborn screening and had negative results (n = 18)	Individual interviews
20	Patterson et al. (2015)	2015	United States	Cystic fibrosis or sickle cell disease	To explore the role of the internet after parents receive abnormal newborn screening results.	Parents who received abnormal newborn screening results and mentioned the internet in their interview (n = 146)	Secondary analysis of existing individual interviews
21	Priddis et al. (2009)	2009	Australia	Cystic fibrosis	To explore the experiences of mother's whose children were diagnosed with cystic fibrosis through newborn screening.	Mothers whose children were diagnosed with cystic fibrosis (n = 19)	Individual interviews
22	Priddis et al. (2010)	2010	Australia	Cystic fibrosis	To explore the impact of cystic fibrosis diagnosis on fathers.	Fathers whose children were diagnosed with cystic fibrosis (n = 15)	Individual interviews
23	Pruniski et al. (2018)	2019	United States	Pompe disease (PD)	To examine the effects of receiving a positive newborn screening result for PD on families	Mothers of children who were diagnosed with PD through newborn screening (n = 9)	Individual interviews
24	Raz et al. (2019)	2019	Israel	PKU, CAH, hypothyroidism, MSUD, homocystinuria, or G6PD	To examines the interface between newborn screening and prenatal diagnosis from the point-of-view of parents of screen positive children.	Parents whose child was screen positive (n = 34)	Individual interviews
25	Raz et al. (2018)	2018	Israel	PKU, CAH, hypothyroidism, MSUD, homocystinuria, or G6PD	To examine the patterns of communication and interaction for peer support among parents of screen-positive children.	Parents whose child was screen positive (n = 34)	Individual interviews
26	Salm et al. (2012)	2012	United States	Cystic fibrosis or hypothyroidism	To examine parents' reactions to newborn screening results and their recommendations for improving communication.	Parents of screen-positive children (n = 106 interviews, 203 parents)	Individual or couple interviews
27	Schmidt et al. (2012)	2012	United States	None	To describe the experiences of families who receive a false-positive newborn screening result in an attempt to discover ways to help improve the newborn screening communication process for families.	Parents whose children (ages 6–16 months) underwent follow-up testing after newborn screening and whose follow-up test results indicated that the newborn screening result was a false-positive (n = 27)	Individual interviews and focus groups
28	Schwan et al. (2019)	2019	United States	X-linked adrenoleukodystrophy (ALD)	To examine the impact of a positive newborn screening result for ALD on families.	Mothers of children who were identiﬁed via newborn screening for ALD (n = 10)	Individual interviews
29	Timmermans and Buchbinder (2010)	2010	United States	Metabolic conditions	To examine how parents and clinical staff work out the social significance of uncertain newborn screening results.	Families of children who visited metabolic genetic disorder clinic, 24 families had ‘deeply ambiguous' diagnosis (n = 55)	Ethnographic observation, individual interviews
30	Tluczek et al. (2006)	2006	United States	Cystic fibrosis	To understand parents' perceptions of genetic counselling while awaiting their child's sweat test results.	Parents of children who had at least one CFTR mutation at time of sweat test (n = 31 couples and 2 single mothers); 25 false positives, 8 true positives	Individual or couple interviews
31	Tluczek et al. (2009)	2009	United States	Cystic fibrosis or hypothyroidism	To understand how parents learned about newborn screening and their suggestions for improving the process.	Parents of 100 newborns recruited from four groups: cystic fibrosis diagnosis, cystic fibrosis carriers, hypothyroidism diagnosis, or normal screens (n = 194)	Content analysis of prior individual interviews
32	Tluczek et al. (2010)	2010	United States	Cystic fibrosis	To examine the psychosocial consequences of newborn screening when cases are clinically ambiguous.	Parents of 5 infants who received abnormal newborn screening results with gene mutations (n = 10)	Individual interviews
33	Tluczek et al. (2011)	2011	United States	Cystic fibrosis	To understand parents' perspectives about the psychosocial consequences of false-positive newborn screening results for cystic fibrosis.	Parents of children who had false-positive screening results for cystic fibrosis (n = 87)	Individual or couple interviews
34	Ulph et al. (2011)	2011	United Kingdom	Haemoglobin disorders	To explore the origins and content of service users' prior knowledge of universal antenatal and newborn screening for haemoglobin disorders.	People who used antenatal and newborn screening for haemoglobin disorders (n = 37)	Individual interviews
35	Ulph et al. (2014)	2014	United Kingdom	Cystic fibrosis or sickle cell disease	To examine parents' intentions to inform their child of newborn screening carrier results.	Family members of children who received a carrier result following newborn screening (n = 67)	Individual interviews
36	Ulph et al. (2015)	2015	United Kingdom	Cystic fibrosis or sickle cell disease	To explore parents' responses to receiving sickle cell or cystic fibrosis carrier results for their child following newborn screening.	Family members of children who received a carrier result following newborn screening (n = 67)	Individual interviews

**Fig. 2:**
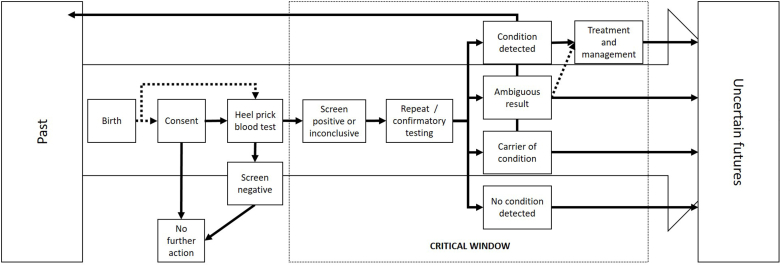
Newborn screening pathways.

Newborn screening has become an embedded part of the neonatal experience for parents. Drawing on the included studies, we mapped the various pathways parents and their children might take when experiencing newborn screening [see Fig. 2]. The newborn screening journey begins in the days after birth, although the screening window ranges from 48 hours (e.g., United States) to up to eight days (e.g., United Kingdom, although most commonly completed on day 5). The consent process varies across, and even within, countries. In the United States, for example, screening is compulsory across nearly, but not all, of the 50 states. In other countries, parents are nominally required to consent to newborn screening. For example, in the United Kingdom under the National Health Service guidelines, healthcare professionals should offer parents screening, and parents may verbally agree (Public Health England, 2018). In practice, however, the extent to which parents are aware of their ability to refuse newborn screening is unclear, as a mother whose child screened negative reported, “It's a very, very quick process and you're not given any option to think about it” (Nicholls, 2012, 300).

Regardless of the differences in the consent process, our review suggests that newborn screening is poorly understood, and its potential ramifications are not readily considered by parents. Parents frequently report not recalling the consent process, or much about the purpose of the screen (Nicholls, 2010, 2012; Nicholls and Southern, 2013; Parsons et al., 2007). Parents describe putting their trust in the healthcare system and medical authority, with newborn screening being largely seen as something that ‘just happens’ after having a baby, rather than an active choice. As one mother of a screen-negative child said, “They obviously know what they're doing, they're trained professionals and they wouldn't just … I couldn't see them doing it just for something to do” (Nicholls and Southern, 2013, 5). For these screen-negative families, newborn screening is ushered in by trusted medical authorities and is typically an experience that passes with little concern or complication.

While the majority of families exit their newborn screening journey swiftly, there is a subset of families who will receive the news that their child screened positive and these families are offered further testing. This is a moment of no return for many families which has implications not only for the infant and parents, but for the family writ large. Before receiving the news, parents may not have realised or appreciated how much impact the “heel prick” test could have on their lives. Most parents – unless they are known carriers or are living with a condition – tend to have limited knowledge of the various screened-for conditions (Chudleigh et al., 2016). This is exacerbated by the fact that for the vast majority of conditions identified through the newborn screening “heel prick”, the infant is typically asymptomatic at the point a positive result is received. However, sometimes non-specific symptoms (such as poor feeding or “failure to thrive”), may have already been observed by the parent(s) (Tluczek et al., 2010). Regardless of context or condition, such news ushered families into a compressed, critical window of time characterised by waiting periods, strong affective responses, and a need for more focused communication (Buchbinder & Timmermans, 2011b, 2012; Grob, 2006; Timmermans and Buchbinder, 2010; Ulph et al., 2015).

Positive screening results are followed up by the offer of confirmatory diagnostic testing, with the nature of this testing varying by condition. From the result of the diagnostic test, the condition is either confirmed (the screening result was a “true positive”) or ruled out (the screening result was a “false positive”). However, for a subset of families, the results of diagnostic testing are somewhat more ambiguous, indicating either a carrier status or a gene variant for which the link to phenotype is neither clear nor certain. For example, there are over 1500 mutations to the CFTR gene associated with cystic fibrosis, although the role of each in creating disease is not clearly understood. Even in cases where a precise diagnosis is made, the broad spectrum of severities associated with the conditions screened for, combined with lack of experience with symptoms at the point of diagnosis (or potential lack of symptoms), can dramatically heighten uncertainty for parents, despite being presented with the seeming certainty of a confirmed diagnosis.

Newborn screening emerged as a complex, and sometimes contradictory, experience for families who received positive or inconclusive newborn screen results. Frequent oscillations characterised the experiences between seeming certainty and uncertainty, combined with distinct contractions and expansions of time, roles and expectations, as well as conceptualisations of health, illness, and patienthood. As a direct consequence of the incremental and provisional character of information generated by the newborn screening process, there are various junctures in the screening pathway where patients must absorb new, unexpected, or entirely revised health information. These junctures represent significant sites of (re)calibration for families, with impacts extending far beyond the boundaries of the screening trajectory and immediate family unit. In the following sections, we examine these junctures and the importance of *timing* within this critical window.

### 3.2 Assessing parents’ absorptive capacity

Across studies, participants frequently used descriptions and metaphors of “absorption” and “digestion” to describe their processing of screening and testing information. For example, “Your brain is a sponge” (Mother of child with cystic fibrosis, Tluczek et al., 2006, 287) and, “There was just too much at that time to absorb” (Mother of child with cystic fibrosis, Grob, 2008, 1062). These metaphors and descriptions were common and prompted us to apply the concept of “absorptive capacity” to screening contexts. “Absorptive capacity” is a term used widely in management studies to refer to a firm's ability to “recognise the value of new, external information, assimilate it, and apply it” (Cohen and Levinthal, 1990, 128). The term is not commonly used at the level of the individual, but has value as a lens through which to examine and explain parents' ability to process new diagnostic information.

For the subset of families that have a positive or inconclusive newborn “heel prick” screen, the initial affective responses tended to be ones of shock and anxiety (Moran et al., 2007; Salm et al., 2012; Schwan et al., 2019). Parents were not necessarily aware of what they consented to (or did not give consent for), so hearing that their child tested positive ushered in a period of confusion and fear (Moran et al., 2007; Salm et al., 2012; Schwan et al., 2019). Before receiving the news, parents conceptualised their child as healthy and perfect, particularly if they had also gone through the process of antenatal screening without any unexpected screening results. As a parent of a screen-positive child said:

“We really didn't have any concerns. And all of our ultrasounds were really, really good. And so we were unconcerned about any of the genetic stuff, was our general feeling.” (Timmermans and Buchbinder, 2010, 413).

Similarly, parents had to reconcile the fact that their child might not be symptomatic and instead “looked” healthy, yet still had a positive newborn screening test. One mother described getting the result that her child screened positive for cystic fibrosis as “very terrifying you know, and I mean you're sitting there and it's like you're holding what you thought was a really healthy baby” (Grob, 2008, 1060). In such cases, parents' distress limited their ability to absorb information in the moments following the news that their child screened positive and at later points in the diagnostic process. This shock was summarised by a mother who discovered her child screened positive for a metabolic condition, “I couldn't even comprehend anything that she said. I couldn't even function at the moment she was telling me.” (DeLuca et al., 2011, 56).

Absorptive capacity is also dependent on one's prior related knowledge, including familiarity with concepts and language (Cohen and Levinthal, 1990). Based on the studies in this review, we argue that even if parents remember consenting to, or being notified about, newborn screening, they do not necessarily have the tools to understand what it means. Parents' distress, and subsequent inability to absorb information, was augmented by their own unfamiliarity with screened-for conditions and genetics (Chudleigh et al., 2016; Pruniski et al., 2018; Tluczek, 2006; Tluczek et al., 2010), potential conflicts between screening and diagnostic test results, equivocal findings, and prognostic uncertainty. Tluczek (2006) points out that even the language surrounding screening and testing can be fraught with confusion, including the counterintuitive meaning of the terms “positive” and “negative” when describing test results, compared to an everyday conversation where these indicate “good” and “bad” respectively. However, even this interpretation overlooks the complexity of ways these terms can be understood and experienced by families. Indeed, there has been a more recent push from families living with a range of screened-for conditions to employ neutral language in screening contexts that do not pre-empt the parents' reception of the news as either “good” or “bad” which has now filtered into professional guidelines. Nevertheless, the use of inaccessible medical language to describe screening and testing results and processes was a widespread concern across the dataset which in turn impacted on parents' absorptive capacities, as a mother of a child who is a cystic fibrosis carrier commented:

“We like to know that he does or he does not have this, instead of using all these big, giant words that make your head swim. It is confusing because one (test) could be a good thing that he's negative, another one could be a bad thing that he's negative.” (Tluczek et al., 2006, 284).

Such specifics inherent in the language of genetics present frustrating difficulties for families that hinder their absorptive capacity:

“[The endocrinologist] spoke way above our heads, and I mean, we're educated parents you know what I mean? But … it was so hard to understand him at times, and we got the basic gist, and we knew what we needed to do … but I just remember it being very, very frustrating.” (Parent of child with congenital adrenal hyperplasia, Boyse et al., 2014, 438)

Having to grapple with unfamiliar terminology and meanings, as well as the inherent uncertainty of what will happen to one's child, fuels parents' worries and anxieties.

Beyond parents' (un)familiarity, the format of information presentation also influences parents’ absorptive capacity. For individuals to effectively absorb information “it is insufficient merely to expose an individual briefly to the relevant prior knowledge” (Cohen and Levinthal, 1990, 131). Consent, or lack thereof, for newborn screening is taken at a time when new parents are simultaneously exhausted, distracted, and busy caring for their new child. New mothers may also be experiencing postnatal physical and mental health concerns of their own. Any information given about the screen at that point does not seem to be experienced as a choice or an active conversation, but rather a minimal (if any) conveyance of information (Nicholls, 2010, 2012; Nicholls and Southern, 2013; Parsons et al., 2007). For the subset of families who are notified of a positive or inconclusive screen, the limited prior communication about the potential implications of newborn screening sets the stage for what is often perceived to be a period of problematic communication (Boyse et al., 2014; Buchbinder and Timmermans, 2012; Locock and Kai, 2008; Salm et al., 2012; Tluczek et al., 2009; Ulph et al., 2015).

By looking across the studies, we were able to consider how parents' information needs varied by condition and context. We identified a continuum of informational needs, with parents preferring different levels of information based on their absorptive capacities at that time. Some parents want to dive in and “consume” as much information as possible, while others want “bite-sized” chunks, others still want to hold back from obtaining information until there is diagnostic clarity. Unfortunately, parents’ needs were not always met. In some cases, parents reported being given a volume of information that they were ill-prepared to receive and unable to absorb. As one mother of a child who screened positive for cystic fibrosis said:

“It was kind of like going back to school. I was trying hard not to fall asleep. I was like, ahh, just so much information tossed at you at one time, you know, your brain is a sponge, but it can only hold so much” (Salm et al., 2012, 373).

In other cases, parents reported wanting more information than was given by their healthcare providers. As a result, they sought other sources – primarily the internet or support organisations – in an attempt to increase their knowledge (Carpenter et al., 2018; Patterson et al., 2015; Raz et al., 2018). As one father of a cystic fibrosis carrier child said, “I want all the information. That way I know what I'm waiting to hear about … And, in fact, we were even seeking it out” (Tluczek et al., 2006, 284).

Informational needs become more complicated in instances where health care providers themselves, who are looked to as the experts, do not have answers (Carpenter et al., 2018; Johnson et al., 2019; Pruniski et al., 2018; Schwan et al., 2019). Healthcare providers may be counselling or treating families with a rare condition for the first time, and this could undermine parents' confidence in the information provided. As one mother said, “The counsellor just gave us a print-out of adrenoleukodystrophy. The specialist didn't know what adrenoleukodystrophy was before Jeremy. We were the ﬁrst” (Schwan et al., 2019, 41). Although we acknowledge that there may be a discrepancy between what healthcare providers say and what parents hear, it seems there is room for improving communication and information provision during this critical window when absorptive capacity is in its highest state of flux.

### 3.3 Newborn screening, uncertainty, and the concertinaing of time

There is a crucial temporal component to parents’ experiences of newborn screening. Throughout the critical window, time expands and contracts, generating ripple effects into the past, present, and future. During the period between a positive screen and (potentially) receiving definitive diagnostic results, families are often living through a state of ambiguity or disorientation. These families experience a compression of time while they are stuck in liminal spaces as “patients-in-waiting”:

“[Inhabiting] a liminal state between normalcy and pathology, imposed by medical screening and testing technologies aimed at secondary prevention, characterised by a lengthy process of medical surveillance to resolve diagnostic uncertainty, which may spill over into personal identity and other areas of life”. (Timmermans and Buchbinder, 2010, 419).

We found that this definition held across papers included in this synthesis, yet how people approached living in the liminal space during the critical window varied considerably.

Families who view ambiguity as a negative state desire definitive answers as a means of gaining control. Such families may want to find out as much as possible about a potential condition and turn to information seeking online and those who have lived experience with a condition in the interim period between a positive screen and receiving diagnostic results (DeLuca et al., 2011; Patterson et al., 2015; Tluczek et al., 2010). Once these families received a diagnosis, they were able to make sense of the condition and how to manage it in the future (Chudleigh et al., 2016). At the same time, parents whose child is living with a screened-for condition may find it exhausting to have to educate others about the condition frequently, as they act as conduits of information for others. While they appreciate the certainty a diagnosis brings, they may seek to minimise the impact such condition has on their lives (Carpenter et al., 2018). Even among those with an ambiguous diagnosis, there may be a drive towards labelling as an attempt to gain control and make the situation more concrete, as explored in a study of families whose children screened positive for cystic fibrosis but had an inconclusive diagnosis (Johnson et al., 2019). For these types of families, knowledge about the condition allows them to feel prepared to manage the health and other needs of their infant moving forward. However, such families must still live with the uncertainty of exactly how the condition will manifest in their child as an individual, and hold out hope that they will not need to use the information in the future.

While some families may seek to learn as much as possible to gain control, other families may view the inherent ambiguity of this liminal period in a more positive light. In such cases, families may not want much information, holding out hope that they may never need to know much about the screened-for condition (DeLuca et al., 2011; Tluczek et al., 2006). These families may adopt a “wait and see” approach to the period between screening positive and receiving a diagnosis, thinking that, “too much information can be hurtful in a sense” (DeLuca et al., 2011, 57). If they are notified that the initial “heel prick” result was a false positive, such families will have avoided the anxiety and stress that additional information could bring. However, if the child is diagnosed with a condition, these families may opt for “‘easy-to digest’ information and ‘just the facts, because you can't handle anything else’” (Boyse et al., 2014, 438), reflecting again the previously discussed concept of absorptive capacity. These families may also continue to look towards the future as a coping mechanism, hopeful that perhaps the condition will not manifest or be of limited severity, or perhaps that treatments will improve over time.

While families may take differing approaches to move through the critical window, they share a common experience that has long-term ramifications for everyone involved. Indeed, for screen-positive families, this experience has ripple effects that stretch out both backwards and forward in time. If it is an inheritable condition, families who receive a positive or ambiguous diagnosis may find themselves looking backwards in an expansion of time, trying to work out where the trait might have come from in the family tree (Chudleigh et al., 2016; Tluczek et al., 2010, 2011). As one parent reflecting on being a carrier for cystic fibrosis said:

“If you kind of think back on all of your family members and relatives and stuff, and even my side and all the family, we both sat there and said, really can't think of anybody's obviously that had it. . . . So it's like, okay, how many family members on either side are possible carriers?” (Tluczek et al., 2011, 182).

As part of newborn screening, parents may find out that they are carriers of a genetic condition that they did not know about (Buchbinder and Timmermans, 2011b; Locock and Kai, 2008). This biographical disruption in the present may prompt parents to look back at their pregnancy (and potentially even earlier) to consider what they might have done differently; as such, there is a decompression of time. For example, a mother of a child with cystic fibrosis, questioned what would have happened with a different husband, saying, “I started thinking if I'd married someone else, I wouldn't have had a baby with him [Father], but then I wouldn't have him [Baby]” (Chudleigh et al., 2016, 1221). As families look backwards, some grapple with shame, blame, and guilt as they consider what might have been for their child, particularly when a child inherits a condition from their parents (Buchbinder and Timmermans, 2011a; Carpenter et al., 2018; Grob, 2008; Johnson et al., 2019; Tluczek et al., 2010, 2011).

Indeed, the impact of newborn screening flows out from the moment of notification of positive or abnormal results as parents must re-write the life that they thought their child would have. In quick succession, parents must initiate a chain of emotional processing, discussion, and medical appointments, involving not just the child but potentially the parents and other family members, too (Grob, 2006; Timmermans and Buchbinder, 2010; Tluczek et al., 2011). For asymptomatic children, the unintended consequences of this journey for parents is a loss of what should be a happy time with their child in the present. As one parent of a child with an ambiguous screening result said,

“We'll always wonder because of the screening, if we didn't know this [cystic fibrosis mutations], we would just assume he's a healthy kid, but now we have the wonder, ‘Is it CF? Could it be?’ It's taken away a little bit of the joy.” (Tluczek et al., 2010, 216).

Indeed, this loss of time in the present becomes more apparent when compared with families who received a later diagnosis:

“I'm actually grateful for what I had [with a later diagnosis], because I did have my moment in the sun. Even if it was that one day in the hospital with all the guests and the flowers and the balloons and thinking I have this beautiful healthy baby. It wasn't robbed from me from the get-go where other people know right away and they never have that moment in the sun. They always have to be anxious, when is it coming, what's going to happen.” (Grob, 2006, 165).

It seems that, for some, the pre-symptomatic diagnoses ushered in by newborn screening limits the joy families might feel in the present. As such, receiving a positive or ambiguous newborn screen effectively acts to extend the impact of the condition.

Newborn screening also has ripple effects that extend into the future and across generations. As families move away from receiving a diagnosis, time expands into uncertainty and the unknown. Parents of children living with conditions will have to consider how, when, and to whom they disclose their child's condition (Johnson et al., 2019). Parents may also consider their own future reproductive intentions, including whether or not they want to become pregnant again and, if so, what role antenatal or newborn screening may play in any subsequent pregnancies (Raz et al., 2019). Parents of children who are carriers of a condition will need to consider if, when, and how they will tell their child about their carrier status (Ulph et al., 2014). One parent considered what might happen if their child was a cystic fibrosis carrier:

“We were talking about when would we tell her. Like, when she was engaged and married? It would almost be too late. If she really wanted to know, I feel like we want to tell her but I can't… really imagine that point in our life yet.” (Tluczek et al., 2011, 180).

These planning conversations take place even as their children are newborns despite recognition that the act of telling may not take place for years to come. Looking further into the future, as the children will also have to consider how they manage their condition or carrier status, how to disclose it to others, and their reproductive intentions (Carpenter et al., 2018).As one mother of a cystic fibrosis carrier said, “He is going to know when he gets older and then that will be up to him how he, what he wants to do. It is up to him if he gets in a serious relationship, if he and his partner want to have children, then obviously you know, she wants to be tested, then that is up to them.” (Ulph et al., 2014, 413).

For some families, this expansive future uncertainty could be met with worry, as the residual risks of future possibilities will not go away over time. Parents who experienced a false positive newborn screen may continue to have residual worry (Schmidt et al., 2012). For parents of children who are carriers of a condition, residual risk is carried forward into the future. One parent of a child who is a cystic fibrosis carrier explained, “As of right now, it [child's carrier status] doesn't mean anything. But, you know, there's always that slim, slim chance that somewhere down the road … something in the human biology where things may change” (Tluczek et al., 2011, 178). These parents know that their child is healthy, yet keep the condition in the back of their mind. Parents of children who have a condition must contend with uncertain futures where the condition worsens:

“I try not to think about the future too much because it freaks me out. I try to envision positive things, but if I think too far in the future sometimes my mind goes: What if he's in a wheelchair? What if he's dead? So I really don't think about it very often. I guess that's the way that I deal with it.” (Parent of child with x-linked adrenoleukodystrophy, Schwan et al., 2019, 42).

At the same time, parents may construe the unknown future as a hopeful state, where perhaps medical treatments improve, and their child's condition never manifests or worsens. As one mother of a child with x-linked adrenoleukodystrophy said, “In 10, 20 years there may be a medication. Or the gene therapy is going to be the gold standard and we're good. I do feel lucky that he is so young and we have time on our side” (Schwan et al., 2019, 42). As such, the implications of newborn screening play out in the months, years, and even decades following diagnosis, suggesting that the aspects of screening are enduring over the life course.

## 4 Discussion

We draw on 36 qualitative studies published over a 20 year period to synthesise qualitative findings about parents' experiences of newborn screening. By looking across the range of moments, outcomes, and conditions that have been captured across international contexts, we have characterised the critical features of the broader newborn screening experience from the family perspective. While currently most families will receive negative newborn “heel prick” screening results, it is also important to consider the experiences of those who receive positive, inconclusive, or ambiguous screening outcomes, particularly as we move into an era of screening using genomic sequencing, which could generate an exponential rise in the number of positive and unexpected newborn screening results for a much larger number of people.

Given the “urgency narrative” often used to promote newborn screening programmes, it can be challenging to critique the expansion of newborn screening panels (Grob, 2019). However, we provide evidence that the experience of screening is variable. We focused on the critical window of time between being alerted to a positive or inconclusive newborn screening result and further testing wherein families must process a range of emotions, determine their informational needs, and shift through alternating periods of waiting and activity. We used the concept of absorptive capacity – the ability to recognise, assimilate, and apply new information – to capture how parents comprehend their child's screening results or condition. We have synthesised evidence to explain the various ways that parents experience the expansion and compression of time throughout and beyond the screening pathway, including potentially far-reaching implications across time, as well as beyond the screened family. Given the complicated pathways involved in newborn screening, future work that takes a reproductive life course approach might more fully capture these ripple effects, potentially shedding light on the longitudinal impact on individuals and families.

The evidence suggests that families experience newborn screening not as a distinct moment in time, but rather as part of a larger journey spanning decades. Knowing this, it is worth thinking about the ways that healthcare services could be improved to better support patients, and healthcare providers, across the newborn screening journey, as suggested by other researchers (Moran et al., 2007; Tluczek et al., 2006, 2009; Ulph et al., 2014, 2015). Returning to the idea of absorptive capacity, the evidence suggests that it is exceedingly difficult for families to absorb the news that their child had a positive or inconclusive newborn screening result. Given that the general public are largely unfamiliar with many of the screened-for conditions, there is a need to prepare prospective parents with information about newborn screening – and the possible implications – early in the journey, during the antenatal period. Indeed, this study supports and strengthens previous calls for newborn screening information provision during the antenatal period, particularly during the third trimester (Kai et al., 2009; Ulph et al., 2017). As screening is initiated rapidly post-birth, when parents are often tired and overwhelmed, the provision of advance information may allow parents the opportunity to better absorb and understand the process, what might happen, and to access support resources. Thus, families will be exposed to the information for a more extended period, potentially become more familiar with the concepts, and might be better prepared to absorb information about screening when the time comes. Additionally, efforts can be made to improve the consent process (where applicable) and communication as the screening journey unfolds. One suggested way of doing this is through the use of personalised information toolkits, although more research is needed in this area (Ulph et al., 2017).

While we focus on the experiences of families, we also recognise that general practitioners and other healthcare providers may not have previously encountered rare screened-for conditions. Thus, they may be unprepared to provide support to families in the immediate aftermath of positive or inconclusive newborn screen results. The evidence we have compiled suggests that the critical window of time between notification of a positive or inconclusive screen and diagnostic results is characterised by intense affective responses that could be better mediated through effective communication and the provision of appropriately tailored information. Equipping healthcare providers with the tools to assess parents' absorptive capacity at various points in their screening trajectory, and the various factors that influence that capacity, could both enable compassionate communication and facilitate the provision of appropriately timed and tailored information that better aligns with the families’ needs.

### 4.1 Strengths & Limitations

We acknowledge that synthesis work is a third-order interpretation of events and that we are far removed from the lives of the people the data represents (Ludvigsen et al., 2016; Sandelowski, 2006). Findings presented here are the result of our collaborative interpretation and shared consensus, which we view as a strength of meta-ethnography but recognise it challenges the epistemological views of some researchers. Relatedly, we acknowledge that there are debates about how best to assess qualitative research included in syntheses (Dixon-Woods et al., 2007). Ultimately, recognising that analysis is carried out for varying purposes and audiences and that qualitative research publications may be constrained by journal word counts, we elected not to exclude any studies based on the quality of reporting. Finally, we based our interpretation on the published work matching our inclusion criteria. We undertook a systematic search to address potential concerns about rigour (Toye et al., 2014), however, we do not aim to make statistical inferences nor summarise the whole body of knowledge about the effects of newborn screening. While the literature was international in scope, it largely came from developed countries; we acknowledge that the experience of newborn screening may differ in other nations not included here. We were particularly interested in examining the experiences of parents undergoing newborn screening, but recognise that many others are part of this picture; future reviews may consider other work involving mixed-methods studies or those centred on health care providers.

## 5 Conclusion

Looking towards the future, the expansion of genomics technologies will likely serve to exacerbate these existing tensions and uncertainties in newborn screening. As access to whole genome sequencing increases, the number of people receiving a positive or uncertain newborn screen will dramatically increase. As such, there is a pressing need to learn from the experiences of people who have already gone through the newborn screening journey. It is critical to question how parents can meaningfully consent to newborn screening for ever wider panels of conditions. Similarly, it is vital to consider people's absorptive capacities – their ability to take on and comprehend-health information about their child. Finally, the long-term implications of screening – both within and beyond the family – ought to be considered. By looking at the experiences of parents, we can anticipate and prepare for these future expansions, which are already occurring in varying degrees across countries.

## Department of Health disclaimer

The views and opinions expressed therein are those of the authors and do not necessarily reflect those of the Health Technology Assessment Programme, NIHR, NHS, or the Department of Health.
